# Endocrinology practice patterns of hypothyroidism and osteoporosis management in a U.S. tertiary academic medical center

**DOI:** 10.1186/s40842-019-0085-8

**Published:** 2019-07-18

**Authors:** Jien Shim, Tiffany Lin, Cody Dashiell-Earp, Meghan Nechrebecki, Angela M. Leung

**Affiliations:** 1 Division of Endocrinology, Diabetes, and Metabolism; Department of Medicine, UCLA David Geffen School of Medicine, Los Angeles, CA USA; 2 Division of Endocrinology, Diabetes, and Metabolism; Department of Medicine, VA Greater Los Angeles Healthcare System, Los Angeles, CA USA; 3 UCLA David Geffen School of Medicine, Los Angeles, CA USA; 4 University of Colorado School of Medicine, Denver, CO USA

**Keywords:** Hypothyroidism, Osteoporosis, Practice patterns

## Abstract

**Background:**

The major published clinical guidelines for the management of hypothyroidism and osteoporosis are not uniformly consistent and may be a significant contributor to variability of clinical care delivered by endocrinologists, in addition to other factors, such as physician experience, physician and patient perceptions, and patient comorbidities. The purpose of this study was to assess practice patterns of hypothyroidism and osteoporosis within an academic endocrine clinic.

**Methods:**

A retrospective medical record review of the first 200 adult patients (*n* = 100 with primary hypothyroidism and *n* = 100 with osteoporosis or osteopenia) seen by an endocrinologist beginning January 2, 2017at a large U.S. urban tertiary academic medical center was performed. Data were collected regarding patient demographics, clinic visit type, patterns of ordering laboratory tests and imaging, and choice of pharmacologic treatment.

**Results:**

Most patients with hypothyroidism (99%) had a serum thyroid stimulating hormone concentration measured. Other thyroid indices measured included serum total thyroxine (10%), serum free thyroxine [T4] (82%), serum free T4 index (6%), serum total triiodothyronine [T3] (9%), and serum free T3 (12%). Forty-eight percent also had serum thyroid antibodies checked. A variety of thyroid hormone supplements were used to treat hypothyroidism, including levothyroxine (83%), levothyroxine and liothyronine combination (8%), and desiccated thyroid extract (6%). In regards to patients with osteoporosis, mean duration of all pharmacologic therapy combined was 73.4 ± 81.9 months. For those with more than one bone density (DXA) scans (64%), the mean time interval between two consecutive DXA scans was variable (mean 32.0 ± 24.7 [SD] months). Sixty eight percent of the patients had bone turnover markers assessed within 7 months of the visit.

**Conclusions:**

This study reports a real-world experience of endocrinology practice patterns at a large U.S. academic healthcare system. For the common diagnoses of hypothyroidism and osteoporosis, there are opportunities for increased standardization of care, particularly regarding the ordering of laboratory testing and radiologic studies. Identifying areas with significant practice variability may improve the quality and health outcomes and reduce the cost of care for patients with these conditions. Increased understanding regarding the reasons behind ordering various studies may help physician and patients further align their goals.

## Background

Together with diabetes mellitus, hypothyroidism and osteoporosis are the most common conditions encountered in a clinical endocrinology practice and contribute significantly toward total U.S. healthcare costs. Hypothyroidism, treated, subclinical and overt, is prevalent in up to 9.4% of the general population [1], and levothyroxine remains the most commonly prescribed medication in the U.S. [2]. In 2008 alone, thyroid disease among women ≥18 years was associated with an estimated $4.3 billion of healthcare expenditures [3]. In addition, approximately 53.6 million adults in the U.S. have osteoporosis or low bone mass, corresponding to 54% of the population age > 50 years [4]. In 2005, there were over 2 million fractures associated with $17 billion in healthcare costs; by 2025, the annual fracture rates and costs are projected to rise by another almost 50% [5].

Published guidelines from several major medical societies on hypothyroidism and osteoporosis are available to provide guidance to providers and standardize care for these conditions (Tables 1 and 2). Recognizing the gaps in evidence based guidelines and reconciling the differences between available guidelines, especially on osteoporosis, are separate unresolved needs [6].

**Table 1:** Societal Recommendations for the Management of Primary Hypothyroidism

Primary Hypothyroidism		
Category	ATA/AACE	Endocrine Society
Laboratory testing	• Recommends measuring TSH, free T4	• Recommends against measuring free T3
Antibody testing	• Consider when diagnosing subclinical hypothyroidism, nodular thyroid disease, pregnant patients, and patients with miscarriages	• No recommendations
Ultrasound	• No recommendations	• Recommends against obtaining an ultrasound, unless a nodule is palpated
Treatment choice	• Recommends levothyroxine monotherapy as standard of care • Recommends against treatment with desiccated thyroid hormone, compounded thyroid hormones, or combination levothyroxine and liothyronine	• No recommendations
Follow up interval	• Laboratory assessment at 4–8 weeks after any dosage change • Laboratory assessment at 6 months, then at 12 month intervals, once on adequate replacement dose	• No recommendations

**Table 2:** Societal Recommendations for the Management of Osteoporosis

Osteoporosis				
Category	AACE	NOF	Endocrine Society	ACP
Monitoring response to treatment with DXA scanning	• Every 1–2 years	• Every 2 years	• Every 1–3 years	• Recommends against checking during the 5 years of pharmacologic therapy
Use of bone turnover markers	• Consider use to assess compliance and efficacy of treatment	• No recommendations	• Recognizes it as an alternative way of identifying therapy response	• No recommendations
Treatment choice	• Alendronate, risedronate, zoledronic acid, or denosumab for high risk patients • Teriparatide, denosumab, or zoledronic acid for high risk patients unable to tolerate oral therapy • Raloxifene or ibandronate for patients requiring spine-specific therapy	• No specific recommendations	• Alendronate, risedronate, zoledronic acid, ibandronate, or denosumab for high risk patients • Teriparatide or abaloparatide for very high risk patients	• Alendronate, risedronate, zoledronic acid, or denosumab treatment for 5 years. • Recommends against estrogen or raloxifene therapy

The current joint guidelines of the American Thyroid Association (ATA) /American Association of Clinical Endocrinologists (AACE) for hypothyroidism recommend that patients be treated with levothyroxine monotherapy, in preference to desiccated thyroid extracts (DTE) [7, 8]. A serum thyroid stimulating hormone (TSH) measurement is recommended at 4–8 weeks following a levothyroxine dose change. Once patient is on a stable thyroid hormone dose, the guidelines advocate that serum TSH be measured every 6–12 months for routine monitoring. The recommendations also advise the consideration of checking a serum TPO Ab titer in patients with subclinical hypothyroidism, nodular thyroid disease, pregnant patients, and patients with recurrent miscarriages. With regards to thyroid imaging, the Endocrine Society recommends against ordering a thyroid ultrasound in patients with abnormal serum thyroid function unless a nodule is also palpated [9], and the ATA encourages that ultrasound be obtained only in suspected or known thyroid nodules or in the initial evaluation of hyperthyroidism [10, 11].

The recommendations from the National Osteoporosis Foundation (NOF) and AACE recommend pharmacologic therapy in patients with history of a hip or spinal fracture; those with T-scores ≤ − 2.5 at the femoral neck, total hip, or lumbar spine on bone densitometry (DXA); or those with a T-score between − 1.0 and − 2.5 who have 10-year probabilities of hip fracture ≥3% or of a major osteoporosis-related fracture ≥20% based on the Fracture Risk Algorithm (FRAX®) [12, 13]. In addition to maintaining adequate calcium and vitamin D intake, pharmacologic treatment with bisphosphonates, receptor activator of nuclear factor kappa-B (RANK) ligand inhibitor (denosumab), or parathyroid hormone 1–34 (teriparatide) is recommended in these patients. The recently published Endocrine Society guidelines recognize abaloparatide (parathyroid hormone related protein analog) as a treatment option for very high risk patients [14]. Recommendations from the Endocrine Society and AACE state that bone turnover markers can be used as a monitoring tool, and that DXA scanning should be performed every one to 3 years to monitor treatment efficacy and compliance [13, 14]. In contrast, the American College of Rheumatology recommends against performing DXA scans more often than once every 2 years [15], while the American College of Physicians (ACP) advises against DXA monitoring during the initial 5 year period of osteoporosis pharmacologic therapy [16].

As there are discrepancies in the major published clinical guidelines for the management of osteoporosis, some variability in clinical practice is expected. However, patterns of substantial deviation from established guidelines may adversely impact patient outcomes and increase healthcare costs. This study aimed to assess the practice patterns among endocrinologists managing patients with hypothyroidism and osteoporosis. The goal was to identify significant variations in practice patterns and target these areas for increased standardization, providing opportunities for improved quality of care regarding these common conditions.

## Methods

This study assessed patterns of practice variability among endocrinologists at a large U.S. urban tertiary academic medical center, University of California Los Angeles (UCLA) Health, which includes four hospitals and 14 outpatient endocrine sites across the greater metropolitan Los Angeles area. The study was deemed exempt from review by the UCLA Institutional Review Board.

Two hundred adult patients ≥ age 18 (*n* = 100 with hypothyroidism and *n* = 100 with osteoporosis or osteopenia) seen by an endocrinologist within any site of the health system were identified from consecutive clinical encounters containing these diagnoses beginning January 2, 2017. All encounters were all contained within 2017, and 100 for each diagnosis was determined to provide an adequate sampling of practice patterns amongst providers of the same institution. For encounters related to hypothyroidism, all patients were included except those with history of thyroid cancer (for whom management is different from the general hypothyroid population) and those with central hypothyroidism. Pregnant patients were included in the study. For encounters related to osteoporosis or osteopenia, inclusion criteria were history of fragility fracture, T-score ≤ − 2.5, or T-score between − 1.0 and − 2.5 with 10-year hip fracture probability ≥3% or a 10-year major osteoporosis-related fracture probability ≥20% based on FRAX®; there were no exclusion criteria.

Two authors participated in data abstraction, and the data collected was checked for interrater error. Data abstraction from the electronic medical record system included subject demographics, encounter type (new or return visit), and time interval to the next endocrinology clinic visit. For patients with hypothyroidism, data were also collected regarding the etiology of hypothyroidism, serum thyroid function laboratory tests, serum thyroid autoantibody titers, thyroid hormone medications (current and prior), and whether a thyroid ultrasound was ordered and if so, for what indications. For subjects with osteoporosis or osteopenia, data were also collected regarding indications for initial treatment, prior treatments, DXA scan results, time interval between consecutive DXA scans, bone turnover marker (serum osteocalcin and bone specific alkaline phosphatase, serum and urine N-terminal telopeptide of type I collagen) results, and the time intervals between which the bone turnover marker measurements were obtained. Subjects with steroid use were noted as well. In both groups, data were also collected regarding any concomitant endocrine diagnoses.

## Results

### Hypothyroidism

Subjects with hypothyroidism (84% women, 62% Caucasian) had a mean age of 52.6 ± 16.7 [SD] years (Table 3). Eight percent of patients also carried the diagnoses of osteopenia or osteoporosis. The majority (72%) of subjects were seen in return for monitoring of their hypothyroidism. Most (99%) had a serum TSH concentration measured near the date of the clinic visit, while some also had serum total thyroxine [T4] (10%), serum free thyroxine [FT4] (82%), serum free T4 index [FT4I] (6%), serum total triiodothyronine [TT3] (9%), and serum free T3 [FT3] (12%) assessed. Forty-eight percent also had serum thyroid antibodies (45% serum thyroid peroxidase antibody [TPO Ab], 31% serum thyroglobulin antibody [Tg Ab], 10% serum thyroid stimulating immunoglobulin [TSI], and 3% serum thyrotropin receptor antibody [TR Ab]) checked either at the current visit or at any time previously during the evaluation of their hypothyroidism.

**Table 3:** Demographics of patients with primary hypothyroidism (*n* = 100)

Age (years)	
Mean ± SD	52.6 ± 16.7
Gender (n)	
Female	84
Race (n)	
Caucasian	62
Asian	14
Black	5
Other	10
Patient refused to answer	8
Unknown	1
Visit type (n)	
New patient visit	28
Established patient visit	72
Interval between current and next visits (months)	
Mean ± SD	4.6 ± 3.1
Patients with return visit within 6 months (n)	46
Etiology of hypothyroidism (n)	
Hashimoto’s thyroiditis	24
Post-operative	10
Post-ablative	14
Unspecified	52
Presence of other concomitant thyroid conditions (n)	
None	72
Thyroid nodule	23
Goiter	9

Also as part of their workup of hypothyroidism, 33% of patients had a thyroid ultrasound done either at the current visit or anytime in the past. Indications for ordering a thyroid ultrasound included monitoring of known thyroid nodules or cysts (45%), palpable goiter or neck enlargement (12%), hyperthyroidism prior to thyroidectomy (6%), suspected thyroiditis (3%), radiation exposure history (3%), and surveillance monitoring for benign disease following a total thyroidectomy (3%), while no reason was specified for 27% of patients with hypothyroidism.

Hypothyroid patients were managed with a variety of thyroid hormone replacement options, including levothyroxine (83%), combination levothyroxine and liothyronine (8%), and 6% DTE (three Nature Throid, one Westhroid, one Bio-thyroid, and one Armour), while 3% were not managed on any thyroid hormone. Ten percent of patients had tried prior treatment regimens different from their current regimen. Of the 83% currently taking levothyroxine monotherapy, six had previously tried DTE and one had tried liothyronine. Of the 6% currently taking DTE, one had previously tried levothyroxine monotherapy. Of the 8% currently taking levothyroxine and liothyronine combination therapy, two had previously tried DTE.

Of those who were seen in return for monitoring (72%) of their hypothyroidism, the mean interval between the current visit and the following appointment was 4.6 ± 3.1 (SD) months; 64% had a return visit less than 6 months from the current visit. Stated reasons for those seen again within 6 months included a change made to the thyroid hormone dose (11%), ongoing monitoring of other endocrine conditions (9%), pregnancy (6%), symptom assessment (5%), initiation of a new thyroid medication that required monitoring (3%), change in the thyroid medication type or brand (3%), monitoring of serum thyroid function tests (3%), medication compliance issues (2%), planned thyroid nodule fine needle biopsy (1%), planned repeat thyroid ultrasound (1%), or unclear (10%).

We did not identify significant differences in management between male and female patients, other than pregnancy in female patients (6%) resulting in an increased frequency in monitoring.

### Osteoporosis or osteopenia

Patients with osteoporosis or osteopenia (87% women, 73% Caucasian) had a mean age of 69.0 ± 11.5 [SD] years (Table 4). The majority (62%) of patients were seen in return for monitoring of their bone health, which was occasionally in conjunction with monitoring of another endocrine condition. Twenty-one percent of patients were noted to have steroid use, and 29% also carried the diagnosis of hypothyroidism.

**Table 4:** Demographics and practice patterns of patients managed for osteoporosis/osteopenia (*n* = 100)

Age (years)	
Mean ± SD	69.0 ± 11.5
Gender (n)	
Female	87
Race (n)	
Caucasian	74
Asian	6
Black	1
Other	16
Patient refused to answer	3
Visit type (n)	
New patient visit	38
Established patient visit	62
Interval between current and next visits, months (mean ± SD)	4.43 ± 2.85
Patients with follow up within 6 months of current visit (n)	44
Indications for initial treatment (n)	
T-score less than −2.5	49
Fragility fracture	17
Osteopenia with positive FRAX® risk	6
Patients managed on different prior therapies (n)	54
One prior therapy	29
Two prior therapies	18
Three prior therapies	7
Total duration of any prior therapies^a^ (months)	
Mean ± SD	73.4 ± 81.9
Median (range)	48 (0.25–458)
Bone turnover marker(s) checked within 7 months of current visit (n)	68

*DXA* Dual-energy X-ray absorptiometry

*FRAX®* Fracture Risk Assessment Tool

^a^Total duration of therapy with any of the following, alendronate, zoledronic acid, denosumab, teriparatide, ibandronate, risedronate, and raloxifene (*n*=37; unknown in *n*=17)

Indications for initial pharmacologic treatment included established osteoporosis [DXA with T-score less than − 2.5 (49%) or history of fragility fracture (17%)] and osteopenia with high fracture risk based on FRAX® estimates (6%). Fifty-four percent of patients had tried other pharmacologic treatments different from their current treatment; 29% had tried one medication, 18% had tried two different medications, and 7% had tried three different medications. The total proportions of prior pharmacologic therapies in the full cohort were: alendronate (42%), teriparatide (13%), risedronate (9%), ibandronate (8%), zoledronic acid (8%), raloxifene (5%), denosumab (4%), and unknown (10%).

Patients seen at the current visit had been treated with pharmacologic therapy (all options combined) for a mean duration of 73.4 ± 81.9 (SD) months, or median 48 (range, 0.25–258) months. For those with more than one DXA scan (64%), the mean time interval between two consecutive DXA scans (the most recent DXA scan at the time of visit vs. either the previous or subsequent DXA scan) was 32.0 ± 24.7 (SD) months. Sixty-eight percent of the patients had bone turnover markers checked within 7 months of the visit.

Of those who were seen in return for monitoring (*n* = 78) of their bone health, the mean interval between the current visit and the following appointment was 4.4 ± 2.9 (SD) months. Among these, 44% had a return visit within 6 months, and stated reasons included ongoing monitoring of other endocrine conditions (24%), monitoring needed after starting initial pharmacologic therapy for their bone health (5%), review of interim biochemical testing for secondary causes of low bone mass (4%), discussion of treatment options following laboratory testing (3%), review of a planned DXA scan (3%), monitoring after completion of dental procedures (2%), and switch of clinicians (1%). Two patients also returned sooner than initially recommended by the clinician.

## Discussion

In this study examining the diagnostic and management patterns regarding two common endocrine diseases, hypothyroidism and osteoporosis, there is observable variability in practice within a large U.S. urban tertiary academic medical center. Our study provides insight and novel data regarding practice patterns in the management of hypothyroidism and osteoporosis. Although practice variability among providers is a well-established phenomenon in clinical medicine [17–22], these findings demonstrate that there may be opportunities for increased standardization of care, particularly regarding the ordering of laboratory testing and radiologic studies, for these conditions.

There are several potential root causes of the practice variabilities observed, which may include factors related to patient preferences and the structure of our current healthcare system (Table 5). Interventions which may help minimize these incongruencies include increasing the availability of evidence-based patient educational materials, implementing clinical decision support tools, utilizing telemedicine and advanced care providers with expertise in specific conditions, implementing proper value-based care model, and developing low-value care metrics. Areas with limited evidence and more uncertainty have more discrepancies between the major societal recommendations.

**Table 5:** Root causes of and potential interventions to decrease practice pattern variabilities

Root Causes	Potential Interventions
Misinformation available to the public and patients	• Increased development of evidence-based patient educational materials
Convenience of ordering a wide variety of diagnostic and monitoring tests	• Implementation of electronic clinical decision support tools based on standards of care
Inefficient use of endocrinologists and/or clinical visit encounters	• Training and greater use of mid-level providers with disease-specific tasks (i.e. skilled nurse practitioners to monitor DXA scans and/or laboratory results between clinical visits) • Deployment of telemedicine to assist with laboratory follow-up not requiring a visit
Fee-for-service system, which favors frequent follow-up visits, radiology studies, and laboratory tests	• Proper implementation of value-based care • Development of low-value care metrics

A recent multicenter study showed that there is significant practice variation in the use of serum thyroid laboratory testing in U.S. academic and nonacademic medical centers [23]. From data obtained across 82 laboratories and 24 healthcare organizations, there were a median of 14 serum FT4 tests, three TT4 tests, four FT3 tests, and two TT3 tests ordered per each 100 serum TSH levels, although the indications for ordering were not ascertained. There can also be substantial variability in the prescribing pattern of thyroid hormone.

The present findings demonstrate some unpredictabilities in the types of serum thyroid function tests and thyroid antibodies ordered, as well as the indications for obtaining a thyroid ultrasound in patients with primary hypothyroidism. There were 82 serum FT4 levels concurrently assessed for the 99 serum TSH levels ordered in patients, the majority (83%) of whom were managed on levothyroxine monotherapy; however, whether the serum FT4 concentrations were ascertained as part of reflexive TSH testing was not assessed. This study highlighted that ordering practices were the most variable when guidelines are not clearFor example, for the patients managed for osteoporosis, a relatively large range was observed in the interval of DXA testing and in whether bone turnover markers were assessed. Although the ACP guidelines recommend DXA testing only after 5 years of osteoporosis treatment, AACE, NOF, and Endocrine Society guidelines recognize DXA testing as a way to monitor therapy [12–14]. Although the evidence is weak, more frequent DXA testing has been associated with improved compliance and may identify those who are noncompliant with therapy or have secondary causes of osteoporosis. Similarly, although evidence is weak, AACE and Endocrine Society recognize bone turnover markers as a way assess response to therapy and to determine duration of drug holiday.

Based on our results, there appear to be potential opportunities to decrease practice variability, particularly regarding overuse of testing, toward the goal of improving health outcomes at a lower cost. The current guidelines for hypothyroidism and osteoporosis diagnosis and management provide some direction in these areas. It has been shown that ordering unnecessary tests is not only costly, but also can pose potential harms by increasing the risks of false-positive results, thus provoking patient anxiety and increasing misdiagnosis and treatment that is not indicated [24–27]. Overtreatment is also of concern, as it can impact opportunity costs to clinicians and patients who may be spending resources on incidental medical findings at the expense of other higher priority needs.

Innovations in healthcare require a demonstration of the relative advantages associated with changes to existing practices [28]. Patient preference and input largely influence the decision-making process. A survey of clinical endocrinologists showed that although an overwhelming majority (99.2%) of providers prefer levothyroxine as the initial treatment for hypothyroidism [29], patients may differentially prefer alternative forms of thyroid hormone replacement [30], as shown in our study that included 83% of patients on levothyroxine monotherapy. Patients may also request specific laboratory tests as a result of widely available misinformation. Some studies have shown that distribution of patient education materials is associated with improved patient knowledge regarding medications and treatment plans, although the results are not necessarily uniform [31, 32]. In addition, ordering laboratory testing is facilitated with electronic health record systems, and poor order menu design can lead to significant overutilization of laboratory tests [23]. There may be opportunities to convert follow-up visits into encounters solely for laboratory review and/or establish a system of alternating appointments with mid-level providers assigned disease-specific tasks or with primary care physicians.

It is important to apply the body of evidence presented in established practice guidelines. There are areas with no clear consensus in society guidelines, where developing clinical decision support can be useful. At UCLA, our division has developed system-wide clinical decision support tools by local consensus for different endocrine diseases. These tools may be even more valuable, but more difficult to implement, when established guidelines are unclear. When integrated electronically, structured note templates or ordering alerts within the electronic medical record system may facilitate practice patterns, as was shown in one study regarding the ordering of unnecessary serum T3 and T4 concentrations [33]. Although excessive ordering notifications can cause “alert fatigue,” if used properly, it can have significant impact in increasing the value of healthcare. In addition, proper implementation of value-based care and development of metrics would decrease low-value care, which are minimally beneficial and may be wasteful and/or even harmful.

Our study is a single-institution study population, but the findings suggest there is variability in the care of patients with hypothyroidism and osteoporosis within a large outpatient endocrine practice. There are inherent differences in each patient situation that should allow for the personalization of medicine. However, inconsistencies in the overall approaches toward patient care may influence the quality of care, healthcare costs, and patients’ perception of treatment choices. In addition, some limitations to this study include the possibility that primary care providers (PCPs) may co-manage these common conditions, in which case PCPs in addition to endocrinologists should be targeted for interventions. Furthermore, practice patterns may have been affected by specific patient characteristics or conditions, concomitant medications, or other factors. Larger, prospective studies are needed to better examine the factors affecting clinical decision-making and the use of routine diagnostic and monitoring tests in these common medical conditions.

## Conclusions

This study identifies areas of clinical practice pattern variability, particularly in the ordering of laboratory tests and radiological studies, within a large U.S. academic endocrine clinic. Several root causes and potential interventions to resolve these issues are presented. Discrepancies between the major societal recommendations, particularly in areas where there are limited evidence and more uncertainty, may be one reason of variable clinical care patterns. Identifying and correction of such areas may improve the quality and health outcomes and reduce the cost of care for patients with these conditions.

## Data Availability

Data sharing is not applicable to this article as no datasets were generated or analyzed during the current study.

## Abbreviations

AACE: American Association of Clinical Endocrinologists
ACP: American College of Physicians
ATA: American Thyroid Association
DTE: Desiccated thyroid extracts
DXA: Bone densitometry
FT3: Serum free T3
FT4: Free thyroxine
FT4I: Free T4 index
NOF: National Osteoporosis Foundation
RANK: Receptor activator of nuclear factor kappa-B
SD: Standard deviation
T4: Total thyroxine
Tg Ab: Thyroglobulin antibody
TPO Ab: Thyroid peroxidase antibody
TR Ab: Thyrotropin receptor antibody
TSH: Thyroid stimulating hormone
TSI: Thyroid stimulating immunoglobulin
TT3: Total triiodothyronine
UCLA: University of California Los Angeles
